# My orthopedic brace inventory (MOBI): a new, reliable, and valid questionnaire to identify barriers to brace adherence in adolescent idiopathic scoliosis treatment

**DOI:** 10.1007/s43390-025-01074-3

**Published:** 2025-03-20

**Authors:** Omar Elsemin, Marie Beauséjour, Justin-Pierre Lorange, Samuel Sassine, Jean Théroux, Soraya Barchi, Julie Joncas, Sylvie Le May, Carole Fortin, Carl-Éric Aubin, Stefan Parent, Nikita Cobetto, Marie-Claire Ishimo, Hubert Labelle

**Affiliations:** 1 Sainte-Justine University Hospital Research Center, Montreal, QC Canada; 2 Department of Community Health Sciences, Faculty of Medicine and Health Sciences, Université de Sherbrooke, Longueuil, QC Canada; 3 Department of Surgery, Faculty of Medicine, Université de Montréal, Montreal, QC Canada; 4 Faculty of Nursing, Université de Montréal, Montreal, QC Canada; 5 School of Rehabilitation, Université de Montréal, Montreal, QC Canada; 6 Polytechnique Montréal, Montreal, QC Canada; 7 Orthopedic Division, Sainte-Justine University Hospital, 3175 Chemin Côte-Sainte-Catherine, Montreal, QC H3T 1C5 Canada

**Keywords:** Adherence to treatment, Orthopedic brace, Adolescent idiopathic scoliosis, Measurement instrument, Validity, Reliability, Health-related Quality of Life (HRQoL)

## Abstract

**Purpose:**

Full-time wearing of an orthopedic brace has demonstrated effectiveness in limiting curve progression in adolescents with idiopathic scoliosis. However, treatment adherence is challenging, with an average wearing time of 13 h/day. Despite this issue, barriers to brace adherence have rarely been studied. The aim of this study was to develop and validate a new instrument tool to evaluate factors influencing brace adherence.

**Methods:**

Our study followed the COnsensus-based Standards for the selection of health Measurement INnstruments criteria (COSMIN). A conceptual framework was initially defined, and experts elaborated, reviewed, and selected candidate items. We also investigated the MOBI’s factorial structure and its psychometric properties.

**Results:**

The MOBI initial version included 32 items related to four conceptual barriers to adherence, namely social/emotional, treatment, patient, and health system/professional. The factorial analysis led to an 18-item inventory with an internal consistency of 0.85 with four better-defined barriers (treatment social/emotional support structure, patient’s self-image and perception, treatment adverse effects, and treatment acceptability. The MOBI-18f correlates with the SRS-22 domain treatment satisfaction and pain and the SF-12 mental health. Patients with poor brace wear time and more severe scoliosis will score higher on the MOBI-18f questionnaire.

**Conclusion:**

The MOBI-18f is a reliable and valid measure of patients’ adherence to brace treatment. This questionnaire can be used to develop interprofessional adherence support intervention in AIS patients undergoing brace treatment.

**Supplementary Information:**

The online version contains supplementary material available at 10.1007/s43390-025-01074-3.

## Introduction

Adolescent idiopathic scoliosis (AIS) is a three-dimensional spinal deformity that affects 1.8% of adolescents aged 10 to 18 years [1–3]. Treatment decisions are based on skeletal maturity and the severity of the curvature [4, 5]. Bracing is the primary treatment for immature patients with Cobb angles ranging from 20 to 40° worn for 20–23 h a day [6]. This approach aims to prevent curve progression and potentially the need for surgery [7, 8]. However, eligible candidates for this treatment must meet specific criteria, including being aged 10–18 years, having a Risser grade of less than 2, and being either pre-menarche or within 1 year post-menarche [9].

The BrAIST study demonstrated that bracing significantly reduces the risk of curve progression to over 50° [10]. In the study, 28% of patients who wore braces experienced progression, compared to 52% of those who received no treatment. However, adherence to the brace is a significant issue, with average wear times reported at only 13 h per day, and reasons for non-adherence are often not reported [10, 11]. Discomfort associated with wearing the brace may include issues like humidity, pressure, limited mobility, and psychological effects such as impacts on self-esteem and social interactions [10, 12].

Health-Related Quality of Life (HRQoL) assessments reveal that AIS can affect various dimensions, including physical, emotional, functional, and social well-being [13–15]. However, few tools [16–18] specifically address brace-related issues or the barriers to adherence identified by the World Health Organization (WHO), which may include health system factors, patient-related factors, disease characteristics, social influences, and treatment-related factors [19]. To address this gap, the MOBI Questionnaire was developed and validated. This tool integrates multiple dimensions to assess HRQoL and treatment barriers faced by AIS patients. This paper outlines the MOBI Questionnaire’s development, validation, and psychometric evaluation.

## Methods

**Fig. 1:**
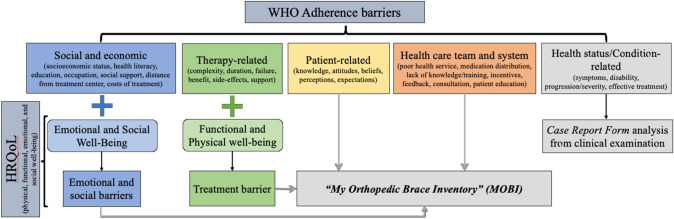
Design process of the MOBI framework

The MOBI tool’s development and validation processes relied on the Consensus-based Standards for the selection of health Measurement Instruments, or COSMIN criteria [20, 21], while considering the five barriers adherence domains provided by the WHO [19] and the four HRQoL domains associated with brace-wearing (Fig. 1).

### Identification of candidate items

A literature review on treatment adherence, the impact of bracing on adolescent quality of life, and related questionnaires was conducted to identify key aspects and develop candidate items. These items were generated by a team of experts, including orthopedists and public health researchers.

A focus group with eight adolescent idiopathic scoliosis (AIS) patients (6 girls and 2 boys) aged 12 to 18 was organized to gather insights on their treatment experiences. This discussion aimed to identify factors contributing to poor adherence and validate points from the literature review. The conversations were recorded and analyzed thematically using QDA-Miner software (v4.1.27). The candidate items were subsequently adjusted based on these findings.

Twelve experts evaluated the refined candidate items for clarity and relevance through a multi-step Delphi process [22], requiring 80% consensus for retention. The questionnaire was then pre-tested with patients to assess item clarity and relevance, leading to further revisions before resubmission to the expert committee.

### Study population for the validation study

The final French-Canadian version of the MOBI was administered to 161 consecutive patients at a metropolitan orthopedic clinic in Canada for scoliosis brace follow-up. Inclusion criteria included a diagnosis of adolescent idiopathic scoliosis (AIS) with a main curve of 20–40° and actively using a TLSO for 22 h daily or having stopped its use for less than 3 months. The exclusions included non-idiopathic scoliosis, cardiac or neurological conditions, syndromic or traumatic origins, and lower limb musculoskeletal anomalies. The questionnaires were administered either on paper or through a secure web-based platform (REDCap) and presented to participants before their meeting with the orthopedist in the waiting area or treatment room. Study coordinators and parents were instructed not to influence the patients’ responses. Informed consent was obtained from the parents, along with assent from the young participants. At that time, the research assistant asked participants to self-report their average daily brace-wearing time.

To assess potential selection biases, participants’ Cobb angle, age, sex, and reasons for declining were anonymously documented and, when available, were compared to participants’ data. The research protocol was approved by the Institutional Review Board (MP-21–2018-1756).

### Factorial validity

The inter-item correlation matrix confirmed the MOBI factorability, ensuring multiple coefficients exceeded 0.3 [23]. The Kaiser–Meyer–Olkin measure evaluating the matrix appropriateness and the anti-image matrix helped verify sampling adequacy, removing items with values below 0.5 [24]. Bartlett’s test of sphericity confirmed that the correlation matrix is not an identity matrix [25]. If factorability is confirmed, exploratory factor analysis using principal axis factoring and applying oblique rotations due to expected inter-variable correlations will be conducted [26, 27]. A comprehensive approach will determine the number of factors, including parallel analysis [28], the minimum average partial method [29], and the scree plot, alongside interpretability and theoretical relevance considerations [30]. Item selection for the MOBI involved analyzing item loadings, cross-loadings, inter-item correlations, floor and ceiling effects, and communality values.

### Reliability

Reliability was assessed through temporal stability and internal consistency. The test–retest method involved participants completing the questionnaire at the scoliosis clinic and again 72 h later from home. Bidirectional mixed effects, absolute agreement, and single-rater inter-class coefficient (ICC) measures were used to compare scores [31], with ICC values interpreting reliability: < 0.5 poor, 0.5–0.75 moderate, 0.75–0.9 good, > 0.9 excellent [32]. Internal consistency was evaluated using Cronbach alpha, where global alpha > 0.8 [33] and > 0.7 for domains were considered good.

### Convergent/divergent validity

Convergent and divergent validity was measured by correlating total scores (Pearson/Spearman) and domain scores of the MOBI, SF-12, and SRS22 questionnaires. A subsample of participants (*n* = 82) was used, hypothesizing that MOBI scores would correlate moderately with SRS22 scores and weakly with SF-12 scores.

### Construct validity: hypotheses testing

Discriminant capacity was evaluated by testing a priori hypotheses on mean differences related to sex, age, Cobb angle, and brace-wearing time. Linear regression, ANOVA, and *t*-tests analyzed these relationships, with *p*-values < 0.05 considered significant. Statistical analyses were performed using IBM SPSSv29 (Armonk, NY: IBM Corp).

**Fig. 2:**
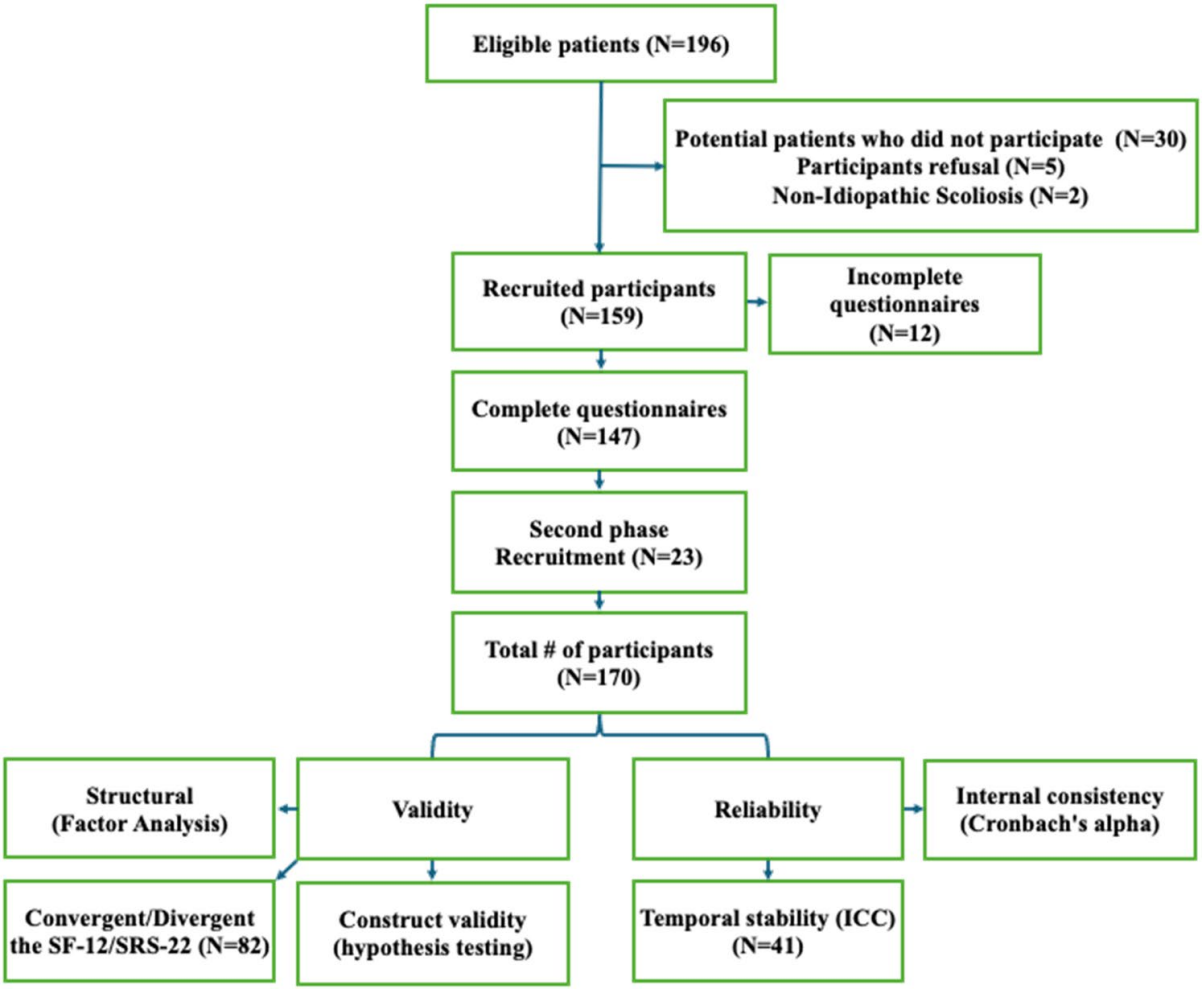
Study participant and recruitment diagram

According to COSMIN criteria [20], 170 AIS patients were targeted for the reliability and validity study. Figure 2 shows the patient flow diagram.

## Results

### Content validity

A team of experts developed an initial questionnaire based on a conceptual framework, literature review, and themes from a focus group analysis that identified barriers to AIS brace treatment adherence. The focus group’s key themes included discomfort, unattractiveness, limitations in social activities, and the importance of support. To ensure clarity and relevance, the items were selected through five Delphi rounds with experts and two pre-tests with 17 patients.

**Fig. 3:**
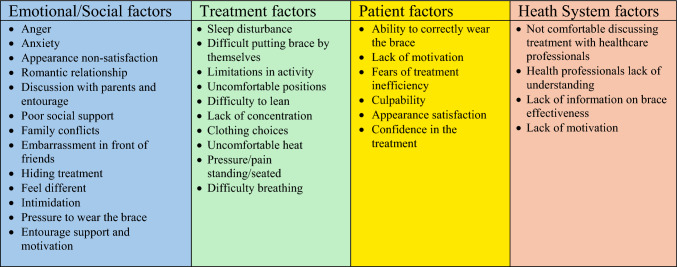
The four conceptual domains influencing poor brace adherence with related items

The final instrument contained 32 items (Appendix 1) categorized into four domains: “Emotional/social barriers,” “Treatment-related barriers,” “Patient barriers,” and “System barriers” (Fig. 3). Responses were recorded on a 5-point Likert scale (0 = Never to 4 = Always), with an additional option for question 13, “impact on love life,” “Not Applicable,” and a modified scale for pain levels. The questionnaire total score is obtained by adding individual item scores. A high score indicated more significant perceived barriers and poorer well-being.

### Participant characteristics

A total of 196 patients were initially identified to complete the IMCO-32 instrument. Of these, 159 agreed to participate and 147 returned completed questionnaires. The recruitment was paused during the pandemic but resumed in May 2022, ultimately increasing the number of participants to 170. Among these 170 participants who completed the IMCO-32, 82 also filled out the SRS-22 and SF-12 questionnaires, while 47 completed the IMCO-32 questionnaire a second time at home 72 h later. The average age of participants was 14 (SD = 2), with 89% assigned female sex at birth. The mean Cobb angle was 30.5° (SD = 9.2), with 51% exhibiting a thoracic curve greater than 30° (Table 1).

**Table 1:** Participants’ demographic and clinical characteristics (*n* = 170)

	Participants *n* (%)
Age (years)	
10 and 13 13 and 16 > 16 years old	48 (28) 97 (57) 25 (15)
Sex	
Girl Boys	146 (89) 24 (11)
Risser (*n* = 169)	
0 1 2 3 4 5	38 (22) 16 (10) 10 (6) 35 (21) 56 (33) 14 (8)
Weight categories according to the WHO guidelines [34]	
Underweight Normal weight Overweight	11 (6) 132 (78) 27 (16)
Main curve’s location and Cobb angle	
Thoracic > 30° Lumbar > 30° Double curve exceeding 30° No curve exceeding 30°	51 (30) 21 (12) 18 (11) 80 (47)
Average SRS-22 participants’ scores (*n* = 82)	
Function Pain Self-image Mental health Satisfaction/Dissatisfaction with Management Total questionnaire	4.0 (0.9) 4.2 (1.0) 3.6 (0.9) 3.9 (1.0) 3.7 (1.2) 3.9 (0.9)

Data from 170 participants regarding MOBI’s 32 items were subjected to a principal axis factoring (PAF) with an Oblimin and Promax rotation. The sample size met the adequacy criteria [26] and the correlation matrix showed several coefficients above 0.3. Two items (20 and 21) were removed due to low correlations on the anti-image matrix.

The Kaiser–Meyer–Olkin measure was 0.843 and Bartlett’s test was significant (*p* < 0.001), indicating suitability for factor analysis. Multiple methods suggested the number of factors to retain: parallel analysis recommended five, while the Scree plot and minimum average partial method indicated four [34, 35]. Ultimately, a four-factor solution was chosen for its interpretability.

Sequential item removal based on communality values and item similarities resulted in an 18-item inventory (Appendix 2) with four better-defined barriers explaining 58.9% of the variance (Table 2). Factor 1 encompassed seven items related to emotional and social support from friends, family, and health professionals, Factor 2 contained four items on self-image, Factor 3 included four items on negative treatment effects, and Factor 4 consisted of three items regarding treatment acceptability. The items’ loadings ranged from 0.37 to 0.96, with a global internal consistency of 0.85 and all domains were above 0.7 (Table 3) [36].

**Table 2:** Factor structure of the MOBI

Items	Factor 1	Factor 2	Factor 3	Factor 4
33	0.960			
31	0.935			
11	0.853			
32	0.787			
29	0.775			
34	0.745			
1	0.605			
7		0.763		
9		0.757		
2		0.561		
6		0.487		
14			0.876	
15			0.742	
19			0.379	
17			0.369	
5				− 0.847
4				− 0.804
16				− 0.429

**Table 3:** MOBI’s internal consistency

Factor	Cronbach Alpha
1	0.75
2	0.76
3	0.74
4	0.76
Complete questionnaire	0.85

The mean MOBI-18f total score was 18.64 (SD: 10.1), ranging from 1 to 46. No ceiling effect on domain scores was found, but a moderate floor effect on the “Health System” and “Social/emotional” barrier domains was observed. A ceiling effect was found on several items, corresponding to positive well-being in many braced patients (Table 4).

**Table 4:** MOBI-18f’s factors mean scores (n = 170)

	Treatment barriers				
	Social/emotional support (item 1 – 13)	Self-image/personal perception (item 14 – 22)	Physical/functional negative effects (item 25 – 30)	Acceptability (item 31 – 34)	Total (item 1 – 32)
Mean score (SD)	1.05 (0,46)	1,52 (0,39)	1,50 (0,46)	0,59 (0,22)	1,21(0,52)
SEM	0,126	0,129	−0,187	0,106	0,0925
% Floor	18.24	7.06	4.70	16.47	
% Ceiling	12.35	10.59	11.76	11.18	

### Reliability

The MOBI-18f inter-rater reliability was good (0.922, 95%CI (0.853–0.958), *p* < 0.001) and fair to good for individual domains (Table 5). A poor degree of reliability was found for two items related to the treatment social/emotional support structure’s domain. Items 32 (ICC = 0.4) (*Are you comfortable talking about your brace treatment with the staff at the scoliosis clinic?*) and item 33 (ICC = 0.39) (*Do you think the staff at the scoliosis clinic informed you enough on the efficacy of the brace?*). All other items demonstrated satisfactory reliability, with the ICC achieving moderate to good values (ICC > 0.5). The standard error of item measurement ranged from 0.29 to 0.79.

**Table 5:** MOBI-18f interclass correlation coefficient

Factors	ICC	95%CI	*P* value
Factor 1 (treatment's emotional/social support structure from family/friends/health professionals)	0,88	0,78 – 0,94	< 0,001
Factor 2 (patient’s self-image and personal perception)	0,70	0,44 – 0,84	
Factor 3 (treatment’s negative effects)	0,63	0,30 – 0,80	
Factor 4 (treatment acceptability)	0,81	0,65 – 0,90	
Total questionnaire	0,92	0,85 – 0,96	

Two-way mixed model with absolute agreement, average measures

*CI* confidence Interval

A correlational analysis found a low, non-significant correlation between the MOBI-18f, SRS-22, and SF-12 total scores (MOBI-18f vs. SRS-22: *r*_s_: 0.17, *p* = 0.124; MOBI-18f vs. SF-12: *r*_s_: 0.10, *p* = 0.37). However, a significant correlation was noted between the MOBI-18f total score and the SRS-22 “Satisfaction with treatment” domain (*r*_s_: 0.24, *p* = 0.031). Additionally, two MOBI-18f domains showed weak but significant correlations with the SRS-22: the “Treatment negative effects physical/functional” domain correlated with “Pain” (*r*_s_: 0.28, *p* = 0.012) and “Satisfaction with management” (*r*_s_: 0.29, *p* = 0.009). The “Treatment acceptability” domain also correlated with “Satisfaction with management” (*r*_s_: 0.23, *p* = 0.042).

Furthermore, the “Treatment negative effects physical/functional” domain correlated weakly but significantly with the SF-12 total score (*r*_s_: 0.27, *p* = 0.015) and the “Mental Health” domain (*r*_s_: 0.24, *p* = 0.03).

### Construct validity (hypothesis testing)

A simple linear regression assessed the relationship between MOBI-18f total scores and self-reported daily brace-wearing hours. The time spent wearing braces significantly predicted MOBI-18f scores (*F*(1,166) = 15.392, *p* < 0.001), accounting for 8.5% of the score variation. Participants adhering to their brace schedule had lower (better) scores.

Participants with scoliosis curves greater than 40 degrees had higher MOBI-18f scores than those with smaller curves, with a difference of −4.096 (95% CI, −7.67 to −0.51), *t*(38.88) = −2.313, *p* = 0.026. No significant differences in MOBI-18f scores were found related to the Risser sign (*p* = 0.58) or sex (*p* = 0.57), and there was no association between age and MOBI-18f scores (*F*(3,633), *p* = 0.58).

## Discussion

We validated the MOBI-18f “My Orthopedic Brace Inventory (MOBI)” (Appendix 2) through factor analysis of the initial 32 items, resulting in a reliable four-factor solution with all coefficients above 0.7 [36]. The questionnaire showed good content validity and temporal stability, meeting COSMIN design criteria [20]. Convergent and divergent validity indicated non-significant correlations with SF-12 and SRS-22 scores, with weak correlations between some MOBI-18f domains and SRS-22 treatment satisfaction and pain. Higher MOBI-18f scores were found in participants with severe scoliosis and poor brace wear time, as expected.

While the SRS-22 [37, 38] and SF-12 [39] are established tools for measuring HRQoL, they do not specifically and holistically address the challenges associated with conservative treatment of AIS and all related underlying problems. The MOBI-18f focuses on treatment barriers rather than overlaps with other HRQoL questionnaires. Although the ISYQoL could have served as a better instrument to assess convergent validity, the French-Canadian version was not available during our study [40]. The MOBI-18f captures the unique impacts of TLSO brace treatment, supporting its construct validity and the relevance of our conceptual framework [41].

Bracing is the most effective conservative method to prevent scoliosis progression [42, 43], yet its success hinges on patient adherence [44, 45]. Unfortunately, observed low brace wear time arises from various functional, psychosocial, and physical factors [46–48]. Thus, finding ways to support adherence is essential for successful treatment.

The MOBI-18f focuses in an original and essential way on the factors influencing adherence to brace treatment and tackles the barriers as defined by the WHO framework, making it particularly suitable for guiding targeted interventions [20]. Unlike existing tools, it offers a comprehensive analysis that goes beyond evaluating health-related quality of life (HRQoL) to uncover the underlying causes of poor treatment adherence. For instance, while the Bad Sobernheim Stress Questionnaire-Brace (BSSQ-Brace) [16] provides an estimate of psychological stress during brace treatment through its concise 8-item structure, its limited validation—particularly in the English version—restricts its reliability and broader applicability. Similarly, the Italian Spine Youth Quality of Life (ISYQOL) [17], despite being grounded in Rasch analysis, dedicates only a small portion of its 20 items to brace-specific challenges, limiting its utility in guiding interventions tailored to brace adherence. The Brace Questionnaire (BrQ) [18], with its 34 items spanning eight quality-of-life domains, offers a broader HRQoL assessment but falls short in granularity; domains with only two items may fail to capture the complexity of patient experiences, and the tool does not address the specific reasons behind non-adherence. In contrast, the MOBI-18f overcomes these limitations by integrating adherence-focused measures and a more robust methodology, providing actionable insights to improve patient outcomes and optimize brace treatment strategies and resources.

The MOBI-18f, validated for clinical use in its French-Canadian version, is designed to identify patients at risk of non-adherence to brace treatment and to support patient-centered interventions. A high score on the MOBI-18f indicates the presence of one or more barriers; thus, instrument domains should be examined to help target specific barrier(s) identified by each of the instrument domains. By aligning with themes of patient involvement and the patient-provider relationship [49], the MOBI-18f facilitates open communication and encourages patients to take an active role in their care. This patient-centered approach is currently being developed at our center [50]. Additionally, we have developed an English version of the questionnaire (MOBI-e), which is currently being validated with an English-speaking population to increase accessibility and expand its use within the scoliosis community.

Our study does have certain limitations. The MOBI-18f was validated in its French-Canadian version, while the English version remains in the process of validation. Furthermore, we used a convenience sample of patients attending regular orthopedic visits during the recruitment period, which could be subject to selection bias. All eligible patients not involved in similar studies were approached, but 30 were not invited due to scheduling constraints, and only five declined to participate. The demographic and clinical characteristics of participants and non-participants were similar, minimizing potential bias. Data collection, which began prior to the COVID-19 pandemic, was interrupted in March 2020, leading to an insufficient sample size for factor analysis at that time. Recruitment resumed post-pandemic, enrolling 23 additional patients, and no significant differences in MOBI-18f scores were observed between pre- and post-pandemic groups (*p* = 0.884). The administration method—completing the questionnaire before the clinic visit and then at home after meeting with the clinical team—may have contributed to the poor reliability of two items that fell below COSMIN criteria [20], though Cronbach’s alpha standards were met and no issues were reported during the questionnaire’s use.

## Conclusion

In conclusion, the MOBI-18f represents a valuable and innovative tool for conservatively managing AIS by addressing critical factors that influence adherence to brace treatment. Validated in its French-Canadian version, it demonstrates strong factorial structure and reliability, making it effective for identifying patients at risk of non-adherence. While construct validity requires further assessment, its potential to support early interventions, enhance patient engagement, and improve treatment outcomes is significant.

The MOBI-18f facilitates patient-centered care by aligning with themes of patient involvement and the patient-provider relationship. Its integration into clinical practice can guide targeted, multidisciplinary interventions and may be paired with self-management tools to improve adherence further. This approach empowers patients to take an active role in their care, leading to better outcomes and optimized resource utilization.

With the ongoing validation of its English version (MOBI-e), the tool’s accessibility and impact will expand, reinforcing its importance in delivering effective, patient-focused AIS management.

## Supplementary Information

Below is the link to the electronic supplementary material.

Supplementary file1 (DOCX 26 KB)

## Data Availability

Measurements and analyses done in the context of this study are hosted at the CHU Sainte-Justine on a password-protected server. Access may be arranged through application to CHU Sainte-Justine Institutional Review Board.
