# Correction: My orthopedic brace inventory (MOBI): a new, reliable, and valid questionnaire to identify barriers to brace adherence in adolescent idiopathic scoliosis treatment

**DOI:** 10.1007/s43390-025-01181-1

**Published:** 2025-09-02

**Authors:** Omar Elsemin, Marie Beauséjour, Justin-Pierre Lorange, Samuel Sassine, Jean Théroux, Soraya Barchi, Julie Joncas, Sylvie Le May, Carole Fortin, Carl-Éric Aubin, Stefan Parent, Nikita Cobetto, Marie-Claire Ishimo, Hubert Labelle

**Affiliations:** 1 Sainte-Justine University Hospital Research Center, Montreal, QC Canada; 2 Department of Community Health Sciences, Faculty of Medicine and Health Sciences, Université de Sherbrooke, Longueuil, QC Canada; 3 Department of Surgery, Faculty of Medicine, Université de Montréal, Montreal, QC Canada; 4 Faculty of Nursing, Université de Montréal, Montreal, QC Canada; 5 School of Rehabilitation, Université de Montréal, Montreal, QC Canada; 6 Polytechnique Montréal, Montreal, QC Canada; 7 Orthopedic Division, Sainte-Justine University Hospital, 3175 Chemin Côte-Sainte-Catherine, Montreal, QC H3T 1C5 Canada

**Correction: Spine Deformity (2025) 13:1075–1084** 10.1007/s43390-025-01074-3

In this article the author’s name Omar Elsemin was incorrectly written as Omar Elsemen. The original article has been corrected.

