# Supplementary material for: My orthopedic brace inventory (MOBI): a new, reliable, and valid questionnaire to identify barriers to brace adherence in adolescent idiopathic scoliosis treatment
Source: Spine Deform. 2025 Mar 20;13(4):1075–84. doi: 10.1007/s43390-025-01074-3 (PMC12227499; doi:10.1007/s43390-025-01074-3)
Supplement: Supplementary file 1 — Supplementary file1 (DOCX 26 KB) [file 43390_2025_1074_MOESM1_ESM.docx]

Appendix 1

| **MOBI-32f Initial Version** | | |
| --- | --- | --- |
| Barriers |  | Items |
| *Barriers link to social and emotional factors  (Barrières liées aux facteurs sociaux et émotionnels  (Barrière sociale émotionnelle) | 1 | **Do you feel comfortable talking about your treatment with your parents and your friends?*  Est-ce que tu te sens à l’aise de discuter de ton traitement avec tes parents et tes amis ? |
|  | 2 | **Do you hide your brace treatment from certain people around you (close friends and family)?*  Est-ce que tu caches ton traitement par corset à certaines personnes de ton entourage ? |
|  | 3 | *Do you think that the people around you (close friends and family) understand how you feel about your brace?*Est-ce que tu penses que ton entourage comprend ce que tu ressens par rapport à ton corset ? |
|  | 4 | **Do you ever have conflicts with your family because of your brace?*  Est-ce qu’il t’arrive d’avoir des conflits avec ta famille à cause de ton corset ? |
|  | 5 | **Do you feel that the people around you (close friends and family) are putting too much pressure on you to wear the brace?*  Est-ce que tu sens que ton entourage te met trop de pression à porter ton corset ? |
|  | 6 | **In general, does wearing your brace make you feel anxious?*  En général, porter ton corset te rend-il anxieux ? |
|  | 7 | **Do you think that wearing your brace lowers your self-confidence?*  Est-ce que tu sens que porter ton corset diminue ta confiance en toi ? |
|  | 8 | **Have you ever received unpleasant comments from the people around you concerning your brace?*  Est-ce que tu as déjà reçu des commentaires déplaisants de la part de ton entourage concernant ton corset ? |
|  | 9 | **Do you feel different from other people around you because of your brace?*  Est-ce que tu te sens différent(e) des autres personnes autour de toi à cause de ton corset ? |
|  | 10 | **In general, does wearing your brace make you feel angry?*  En général, porter ton corset te met-il en colère ? |
|  | 11 | **Do you feel that the people around you (close friends and family) support and motivate you to wear your brace?*  Est-ce que tu sens que ton entourage (amis proches et famille) te soutient et te motive à porter ton corset ? |
|  | 12 | **Have you ever prevented yourself from hanging out with your friends because of your brace?*  Est-ce que tu t’es déjà empêché(e) de faire des sorties avec tes amis à cause de ton corset ? |
|  | 13 | **Do you think your love life is affected by the brace?*  Est-ce que tu considères que ta vie amoureuse est affectée à cause du corset ? |
| *Barriers link to treatment factors  (Barrières liées aux facteurs du traitement lui-même)  (Barrières traitements) | 14 | **Does your brace cause you pain in some areas of your body?*  Est-ce que ton corset te cause de la douleur à certains endroits ? |
|  | 15 | **In general, what is your level of pain when you wear the brace?*  En général, quel est ton niveau de douleur lorsque tu portes ton corset ? |
|  | 16 | **Does your brace prevent you from sleeping well at night?*  Est-ce que ton corset t’empêche de bien dormir pendant la nuit ? |
|  | 17 | **Do you have difficulty concentrating at school when you wear your brace?*  Est-ce que tu as de la difficulté à te concentrer à l’école lorsque tu portes ton corset ? |
|  | 18 | **Does your brace prevent you from doing leisure activities that you like?*  Est-ce que ton corset t’empêche de faire les activités de loisirs que tu aimes ? |
|  | 19 | **Does your brace prevent you from breathing normally?*  Est-ce que ton corset t’empêche de respirer normalement ? |
|  | 20 | **Do you have difficulty putting on your brace by yourself?*  Est-ce que tu as de la difficulté à mettre ton corset seul ? |
|  | 21 | **Does your brace prevent you from wearing the clothes that you like?*  Est-ce que ton corset t’empêche de porter les vêtements que tu aimes ? |
|  | 22 | **Do you feel unbearably warm when you wear your brace?*  Est-ce que tu ressens de la chaleur insupportable en portant ton corset? |

| *Barriers link to patient’s factors  (Barrière liée aux facteurs relatifs aux patientx)  (Barrières patients) | 23 | **Do you feel able to follow the instructions for wearing your brace as prescribed?*  Est-ce que tu te sens capable de respecter les consignes pour porter ton corset tel que prescrit ? |
| --- | --- | --- |
|  | 24 | **Are you motivated to follow your treatment by wearing your brace as prescribed?*  Est-ce que tu es motivé(e) à suivre ton traitement en portant ton corset tel que prescrit ? |
|  | 25 | **Do you feel guilty when you do not wear your brace as prescribed?*  Est-ce que tu te sens coupable lorsque tu ne portes pas ton corset tel que prescrit ? |
|  | 26 | **Are you satisfied with the way you look when you wear your brace?*  Est-ce que tu es satisfait(e) de ton apparence en portant ton corset ? |
|  | 27 | **Are you confident that your brace is controlling your scoliosis?*  Est-ce que tu as confiance en ton corset pour contrôler ta scoliose ? |
|  | 28 | **In general, would you say that your brace improves the comfort of your back?*  En général, dirais-tu que ton corset améliore le confort de ton dos ? |
| *Barriers link to the healthcare system factors  (Barrières liées au système de santé et aux professionnels du traitement)  (Barrière système de santé) | 29 | **Do you think the staff at the scoliosis clinic gives you clear instructions on how to correctly wear your brace? (For example: the doctor, the nurse, the orthotist, the physiotherapist, etc.)*  Est-ce que tu trouves que le personnel de la clinique te donne des consignes claires sur comment bien porter ton corset ? |
|  | 30 | **Are you comfortable talking about your brace treatment with the staff at the scoliosis clinic? (For example: the doctor, the nurse, the orthotist, the physiotherapist, etc.)*  Est-ce que tu te sens à l’aise de discuter de ton traitement par corset avec le personnel de la clinique de scoliose ? |
|  | 31 | **Do you think the staff at the scoliosis clinic informed you enough on the efficacy of the brace? (For example: the doctor, the nurse, the orthotist, the physiotherapist, etc.*  Est-ce que tu penses que le personnel de la clinique t’a suffisamment informé(e) sur l’efficacité du corset ? |
|  | 32 | **Do you feel that the staff at the scoliosis clinic motivates you to wear your brace? (For example: the doctor, the nurse, the orthotist, the physiotherapist, etc.)*  Est-ce que tu sens que le personnel de la clinique te motive à porter le corset ? |
| *The English translation is solely provided to aid readers' understanding of the questions. This questionnaire is currently being validated for use in English-speaking population. | | |

Appendix 2

| Questionnaire MOBI-18f*† | | | | | | |
| --- | --- | --- | --- | --- | --- | --- |
| ***Questionnaire Instructions***  This questionnaire is here to help our care team understand what it's like for you to wear a scoliosis brace.  Remember, this isn't a test—there are no right or wrong answers! As you go through the questions, take a moment to think about how you've felt since you started your scoliosis brace treatment.  Just pick the number that best fits your situation for each question, and feel free to choose only one answer.  Thank you for sharing your experiences with us! | | | | | | |
|  |  | 0 | 1 | 2 | 3 | 4 |
|  |  | Never | Rarely | Sometime | Often | Always |
| 1 | *Does your brace prevent you from sleeping well at night?* |  |  |  |  |  |
| 2 | *Do you have difficulty concentrating at school when you wear your brace?* |  |  |  |  |  |
| 3 | *Does your brace prevent you from breathing normally?* |  |  |  |  |  |
| 4 | *Do you feel that the people around you (close friends and family) support and motivate you to wear your brace?* |  |  |  |  |  |
| 5 | *Do you hide your brace treatment from certain people around you (close friends and family)?* |  |  |  |  |  |
| 6 | *Do you think the staff at the scoliosis clinic gives you clear instructions on how to correctly wear your brace? (For example: the doctor, the nurse, the orthotist, the physiotherapist, etc.)* |  |  |  |  |  |
| 7 | *In general, does wearing your brace make you feel anxious?* |  |  |  |  |  |
| 8 | *Are you comfortable talking about your brace treatment with the staff at the scoliosis clinic? (For example: the doctor, the nurse, the orthotist, the physiotherapist, etc.)* |  |  |  |  |  |
| 9 | *Do you think that wearing your brace lowers your self-confidence?* |  |  |  |  |  |
| 10 | *Are you confident that your brace is controlling your scoliosis?* |  |  |  |  |  |
| 11 | *Do you think the staff at the scoliosis clinic informed you enough on the efficacy of the brace? (For example: the doctor, the nurse, the orthotist, the physiotherapist, etc.* |  |  |  |  |  |
| 12 | *Do you ever have conflicts with your family because of your brace?* |  |  |  |  |  |
| 13 | *Do you feel that the people around you (close friends and family) are putting too much pressure on you to wear the brace?* |  |  |  |  |  |
| 14 | *Do you feel that the staff at the scoliosis clinic motivates you to wear your brace? (For example: the doctor, the nurse, the orthotist, the physiotherapist, etc.)* |  |  |  |  |  |
| 15 | *Do you feel comfortable talking about your treatment with your parents and your friends?* |  |  |  |  |  |
| 16 | *Do you feel different from other people around you because of your brace?* |  |  |  |  |  |
| 17 | *Does your brace cause you pain in some areas of your body?* |  |  |  |  |  |
| 18 | *In general, what is your level of pain when you wear the brace?* |  |  |  |  |  |
| ***The English translation is provided to aid readers' understanding of the questions. This questionnaire is currently being validated for use in English-speaking population.  *†Please contact the corresponding author to obtain a French version of the questionnaire.* | | | | | | |
